# Patient-reported outcome measures for lupus nephritis: content validity of LupusQoL and FACIT-Fatigue

**DOI:** 10.1186/s41687-024-00783-z

**Published:** 2024-09-30

**Authors:** Mona L. Martin, Jennifer N. Hill, Jennifer L. Rogers, Deven Chauhan, Wen-Hung Chen, Kerry Gairy

**Affiliations:** 1 Evidera, Patient Centered Research, Bethesda, MD USA; 2 Duke University School of Medicine, Division of Rheumatology & Immunology, Durham, NC USA; 3 GSK, Value Evidence and Outcomes, Brentford, UK; 4 GSK, Value Evidence and Outcomes, Collegeville, PA USA

**Keywords:** Content validity, FACIT-Fatigue, Lupus nephritis, LupusQoL, Patient-reported outcomes, Quality of life

## Abstract

**Background:**

Lupus nephritis (LN), a severe organ manifestation of systemic lupus erythematosus (SLE), significantly impacts health-related quality of life (HRQoL). Functional Assessment of Chronic Illness Therapy-Fatigue (FACIT-Fatigue) and Lupus Quality of Life (LupusQoL) have been validated to measure HRQoL in SLE, but not specifically in LN. Patient-reported symptoms of LN are not well-reported. We assessed the content validity and relevance of these measures in evaluating patients with LN and their LN-related experiences.

**Methods:**

This qualitative, interview-based study enrolled patients with LN from three US sites from a larger, retrospective survey study. The interview comprised an open-ended concept elicitation part and a more structured cognitive part. Concept elicitation was used to identify relevant themes describing the patients’ experiences. Patients were asked to describe their LN-related symptoms, the severity and impact of those symptoms and their satisfaction with treatment. A cognitive interview approach evaluated the appropriate understanding of the items, instructions, and response options and asked patients about their understanding of the FACIT-Fatigue or LupusQoL measures, their relevance to the condition, and any aspects of confusion or need for better clarity of the questionnaires. All interviews were recorded and transcribed. The concept elicitation data were coded, while the cognitive interview data were tabulated to present the participants’ responses next to the interview questions to support the evaluation of their understanding of the questionnaire items.

**Results:**

Overall, 10 patients participated in FACIT-Fatigue and another 10 in LupusQoL interviews; 18 patients were female, 10 were Black (self-reported) and 17 were receiving maintenance treatment for LN with stable disease activity. When patients recalled their symptoms, 670 expressions of varying symptoms were reported. All patients described pain, discomfort, and energy-related symptoms. Urinary frequency and non-joint swelling were most frequently attributed to LN rather than SLE. Patients felt the questions asked in the FACIT-Fatigue and LupusQoL surveys were relevant to their LN experience.

**Conclusions:**

The symptoms reported by patients with LN were consistent with symptoms reported by the overall SLE population. However, patients indicated that some symptoms of LN were more profound than symptoms of SLE alone, affecting a broad range of areas of daily life activity and resulting in a higher burden on their HRQoL. FACIT-Fatigue and LupusQoL demonstrated content relevance as meaningful tools for patients with LN. However, further quantitative data collection is needed to ensure that these patient-reported outcome tools demonstrate good measurement properties in an LN population.

**Supplementary Information:**

The online version contains supplementary material available at 10.1186/s41687-024-00783-z.

## Abstract (de)

Lupus nephritis (LN) is a kidney involvement in many patients with systemic lupus erythematosus (SLE). SLE patients with LN may have clinical symptoms that include tiredness and changes regarding urine, in addition to those related to SLE itself, like rashes, joint pain and swelling. The symptoms can be mild, moderate or severe and can affect quality of life. It is important to learn the effects of these symptoms directly from the patients; understanding patients’ experiences will help their medical team monitor the disease and treatment more effectively. Questionnaires are often used to evaluate symptoms and the impact of these symptoms on patients’ lives. Two questionnaires, Functional Assessment of Chronic Illness Therapy-Fatigue (FACIT-Fatigue) and Lupus Quality of Life (LupusQoL), were used to assess symptoms and the effect of LN on the patients’ lives. First, patients were asked to describe their signs and symptoms and how these affect their lives. Then, patients were asked about their understanding of the individual questions. We found that all patients reported symptoms of tiredness and joint pain. Increased urinary frequency, swelling in non-joints, and frequent infections were reported more often by patients with LN than those with SLE alone. Patients said that their manifestations greatly affect their everyday lives. There was a good understanding of the questionnaires, and patients felt the questions were relevant to them. This is an important step to validate FACIT-Fatigue and LupusQoL. This study shows that the FACIT-Fatigue and the LupusQoL can be used when assessing people with LN.

## Background

Systemic lupus erythematosus (SLE) is a chronic, autoimmune disease, characterised by immune dysregulation, autoantibody and immune complex formation, and a range of clinical manifestations that can affect multiple organ systems [1, 2]. Lupus nephritis (LN) is a severe manifestation of SLE, occurring in approximately 40% of patients with SLE [3]. Despite therapy advancement, approximately 20% of patients with LN progress to end-stage kidney disease within 10 years of diagnosis, requiring dialysis or renal transplant, increasing early mortality and significantly impacting all aspects of patients’ lives [4, 5].

Health-related quality of life (HRQoL) is a commonly used self-reported measure of a patient’s functioning and well-being across physical, mental and social aspects of health [6]. Although there is a high incidence of LN in patients with SLE, its impact on HRQoL has not been investigated as frequently as HRQoL in the overall population of patients with SLE. Some studies identified a significant burden of fatigue and poor HRQoL in patients with LN [7]. Symptoms such as fatigue, depression and pain are common to both LN and SLE patients and negatively impact a patient’s HRQoL and ability to work [7–10]. Additionally, some studies suggest that HRQoL scores are worse in patients with LN than SLE, specifically the aggregated physical component scores of the 36-item Short Form Survey (SF-36) [11, 12].

While fatigue has been reported as the most burdensome symptom of LN contributing to poor HRQoL [7], other factors may drive poor HRQoL in this patient population. Physical impairments and dissatisfaction with disease management have been shown to contribute to poor HRQoL in SLE [13, 14]. Finally, real-world evidence suggests patients with LN are less satisfied with their current treatments than those with non-nephritis SLE, which may also lead to greater HRQoL impairments.

Multiple HRQoL patient-reported outcome (PRO) measures have been validated for use in patients with SLE [15–18] and used in trials in SLE to assess symptom experience and impacts [19–21]. One such measure is the Functional Assessment of Chronic Illness Therapy-Fatigue (FACIT-Fatigue) questionnaire, which includes 13 items designed to capture physical and mental aspects of fatigue and their impacts [22]. Originally designed for use in cancer, it has been used extensively and has been validated for measuring fatigue severity in patients with SLE [23]. Another validated measure is Lupus Quality of Life (LupusQoL), comprising 34 items across 8 domains (physical health, pain, planning, intimate relationships, burden to others, emotional health, body image and fatigue), which assesses the impact of SLE on HRQoL from the patient’s perspective [24]. While FACIT-Fatigue and LupusQoL have been used in previous studies of LN, including a real-world study [13] and ongoing clinical trials [25–27], these measures have not been validated specifically for LN.

To evaluate how well these measures reflect all important aspects of a concept of interest, cognitive interview methods, which are an accepted approach for assessing content validity [28], were used to assess the extent to which the measures effectively assess the concepts most significant and relevant to a patient’s condition and its treatment. All items within a measure should be relevant to the concept of interest within a specific population and context of use. Comprehensiveness requires that no key aspects of the concept are missing. Comprehensibility requires evidence that the items are understood by patients as intended by the developers of the measure [29]. Evaluation of the content validity of the FACIT-Fatigue and LupusQoL measures is necessary as patients with LN may differ from the overall SLE population regarding their experience with the symptoms and impacts of their condition, and their perceived burden [13, 14]. Qualitative evidence and content validation are vital to ensure these two PRO measures fully capture the patient experience and support their use in clinical trials [30]. This qualitative study aimed to evaluate the relevance and content validity of FACIT-Fatigue and LupusQoL measures in patients with LN, in addition to describing the patient-perceived experience with the symptoms and impacts of LN, and their experience and satisfaction with therapies.

## Methods

### Study design

This qualitative interview sub-study was conducted between December 2020 and November 2021 in patients with LN recruited from three sites in the USA (New York, Texas, and North Carolina) that were participating in a larger, retrospective chart review and survey study. Patients were approached during the main chart review study and invited to participate in this qualitative interview sub-study. Those who agreed to participate in the sub-study were enrolled until the predetermined sample size of 20 was attained and enrolment was complete. Further details of the main study methodology are presented in Supplementary materials.

### Patient population

Patients eligible for this interview sub-study were adults with LN, either biopsy-proven (class III ± V, VI ± V, or pure class V) or suspected proliferative/membranous LN requiring immunosuppressive therapy if diagnosis occurred after March 2020 (biopsy confirmation was not possible due to the coronavirus pandemic). Patients were determined by site staff to be competent to complete a 90-minute interview in US English and to consent to medical record access. Full eligibility criteria of the main study are presented in Supplementary materials.

All recruitment, screening and consenting activities were pre-approved by the relevant Institutional Review Boards. All data were kept strictly confidential in accordance with local, state, and federal regulations and International Council for Harmonisation Good Clinical Practice guidelines.

A sample size of 20 patients was targeted to reach saturation of concept, the point at which new information is no longer being obtained. Once achieved, it is not likely that additional interviews would produce new information. This estimate was based on a previous qualitative work that evaluated the content validity of the FACIT-Fatigue for use within the SLE population [16].

### Interview process and development

A two-part semi-structured interview guide was developed, in accordance with best practice methodology for establishing content validity [31, 32], to cover the concept elicitation and cognitive interview sections for the FACIT-Fatigue and LupusQoL. To manage the overall patient burden in the interview process, ten patients were allocated to each group to participate in either the FACIT-Fatigue or LupusQoL interviews. Patient interviews were conducted via teleconference using Microsoft Teams by four experienced qualitative research staff from Evidera, with extensive expertise in PRO measurement science. The interviewers of this study were expert interviewers who had received additional training regarding aspects of LN, the specific needs of the study, and had been oriented to the details and structure of the interview guide and key aspects of the interview. Two interviewers were also involved in developing the interview guide.

Concept elicitation focused on LN symptoms and impacts as part of patients’ disease experience, and their satisfaction with treatment. This section of the interviews included open-ended questions, allowing patients to describe their symptoms spontaneously, with follow-up probes and verbal rating exercises to obtain the patient’s ratings of severity of individual symptoms (0–10 numerical rating scale [NRS], where 0 indicated no severity and 10 indicated extreme severity) and symptom-related bothersomeness (0–10 NRS, where 0 indicated no bothersomeness and 10 indicated extreme bothersomeness).

Cognitive interviews consisted of think-aloud portions while the PRO was completed and questions focused on the patient’s perception of relevance and comprehensiveness, their ability to understand the items of either the FACIT-Fatigue or LupusQoL PROs, and their ability to understand the response options and use them to select a meaningful answer. Additionally, patients were asked whether there was any concept missing from the questionnaires that they deemed important for capturing their experience accurately. Patients were also asked if they felt the questions in the PROs were relatable to their experiences with LN.

Each PRO item was displayed on the screen by the interviewer and patients provided answers to the questions verbally, which were noted by the interviewer.

### Data collection and analysis

Patient characteristics data were collected as part of the main study through electronic case report forms and are presented descriptively (mean, standard deviation [SD], median, interquartile range [IQR]). All interviews were recorded and transcribed verbatim by a vetted commercial transcription company, following contractual instructions for the task and deliverable. To ensure consistency and quality, all interviewers had one of their early interview audio files evaluated by a senior interview trainer against a list of core competency criteria [31]. Each transcript was reviewed by the Evidera team to identify and redact any personally identifying information that was inadvertently mentioned by the participant during the interview. The clean transcripts were then loaded into the ATLAS.ti software (version 9.0.24.0) to track the assignment of codes to concepts patients talked about in the open-ended part of the interview. Cleaned transcripts were also used to extract quotations from the cognitive interview questions for tabulation.

Initial concepts were defined in a preliminary coding framework based on study objectives and known aspects of SLE and LN. New codes were established as new concepts were identified in the transcripts. The concepts identified were organised into subdomains based on similarity of content or theme within the coding framework, which served as a structured way to organise and classify the interview data. Concepts were organised by either symptom concepts based on patient-reported symptoms of SLE and LN or impact concepts based on the impacts of these symptoms on patients’ lives. To support evidence of appropriate patient understanding of the item content, patient responses to the specific interview questions asked in the cognitive portion of the interview were evaluated for evidence of appropriate comprehension or aspects of confusion regarding the meaning of items. Thematic analysis was conducted to identify content or theme-based relevance between patient expressions and PRO content. Consistency in the coding of concept elicitation data was evaluated by independent dual coding of a randomly selected 10% of the final transcripts. The dual-coded transcripts were compared for percentage agreement between the codes assigned (inter-coder agreement). Coders were experienced research staff who were further trained in the coding process for this study and monitored closely by the qualitative data manager during the coding process. Resolutions of any discrepancies were decided by the qualitative data manager and communicated to the coders for clarity in subsequent coding.

To assess the saturation of concept, the interview transcripts were organised chronologically into five groups of four transcripts each. Newly appearing concept codes in each transcript group were identified in columns as ‘first mention’ of the concept.

## Results

### Patient population

Overall, 20 patients with LN in the USA were recruited into this sub-study and completed interviews. Concept elicitation portions of the interview lasted 30–45 min and cognitive interview portions lasted 30–60 min. Clinical characteristics were available for 19 patients as one patient was missing data. Mean (SD) age at time of LN diagnosis was 27.6 (11.6) years (Table 1). The physician-reported mean time since diagnosis was 14.2 years for SLE and 7.7 years for LN; though, during interviews, patients reported longer times since diagnosis of 15.6 and 11.0 years, respectively.

**Table 1:** Patient demographics and clinical characteristics (collected as part of the main study questionnaire)

	***N*** ** = 19** ^**1**^
**Age (years)**	
Mean (SD)	35.2 (11.0)
Median (IQR)	30.0 (27.0, 48.0)
**Female**,*** n* (%)**^**2**^	18 (94.7)
**Race**,*** n* (%)**^**3**^	
White	3 (15.8)
Black	10 (52.6)
Other	1 (5.3)
Missing	5 (26.3)
**Ethnicity**,*** n* (%)**^**3**^	
Hispanic or Latino	1 (5.3)
Not Hispanic or Latino	13 (68.4)
Missing	5 (26.3)
**Education level**,*** n* (%)**^**3**^	
Primary	0
High school	3 (15.8)
College or university	7 (36.8)
Graduate school	4 (21.1)
None	0
Prefer not to say	0
Missing	5 (26.3)
**Age at LN diagnosis (years)** ^**2**^	
Mean (SD)	27.6 (11.6)
Median (IQR)	25.3 (19.0, 40.5)
**Time since SLE diagnosis (years)**,** median (IQR)**	11.2 (9.1, 19.5)
**Time since LN diagnosis (years)**,** median (IQR)**	6.7 (2.8, 11.2)
**Proteinuria level ≥ 1 g/day or equivalent by uPCR**,*** n* (%)**^**4**^	2 (10.5)
**Experiencing a renal flare at enrolment**,*** n* (%)**	1 (5.3)
**Renal function stable at enrolment**,*** n* (%)**	17 (89.5)
**Treatment phase**,*** n* (%)**	
Induction of renal response with IV CYC or MMF/MPA	2 (10.5)
Maintenance of renal response	17 (89.5)
**LN classification**,*** n* (%)**	
Class III	4 (21.1)
Class III + V	1 (5.3)
Class IV	6 (31.6)
Class IV + V	1 (5.3)
Class V	7 (36.8)
Class VI (+/- V)	0

^1^One patient was missing demographic data; ^2^data reported from the eCRF; ^3^data reported from patient questionnaire; ^4^documentation available at patients’ most recent visit

*CYC* cyclophosphamide; *eCRF* electronic case report form; *IQR* interquartile range; *IV* intravenous; *LN* lupus nephritis; *MMF* mycophenolate mofetil; *MPA* mycophenolic acid; *SD* standard deviation; *SLE* systemic lupus erythematosus; *uPCR* urinary protein: creatinine ratio

The most common LN classification was class V (36.8%), followed by class IV (31.6%), class III (21.1%), class III + V and class IV + V (both 5.3%).

Most patients (89.5%) were receiving maintenance treatment for LN and had stable renal function (89.5%). Two patients (10.5%) had proteinuria ≥ 1.0 g/day; one patient was considered by the physician to be experiencing a renal flare at screening.

### Quality of data

The inter-rater agreement between the three coders was high: 89.3–92.6% for the identification of concepts, and 86.4–98.3% for coding of each symptom. Regarding saturation of concept, 93 concepts were expressed (63 symptom concepts and 30 impact concepts); 48 (51.6%) concepts appeared in the first group of transcripts, 20 (21.5%) in the second group, 10 (10.8%) in the third group, 7 (7.5%) in the fourth group, and 8 (8.6%) in the fifth group. While some concepts appeared in the fifth group of transcripts, the symptoms were very specific (e.g., discoid scars, skin infections and skin scars), each falling within the symptom categories covered in previous groups (e.g., skin symptoms). As such, these specific symptoms were not considered as new concepts but rather as more detailed descriptions of concepts that had already been expressed, using different language. As 91.4% of concepts emerged by the 16th interview (i.e., by fourth group), these results suggest that the sample size of 20 patients was acceptable to reach concept saturation and additional interviews would not have been likely to produce additional new concepts.

### Symptoms of LN

Patients expressed a wide range of symptoms. Patient-reported symptoms and their relationship with SLE, LN or both, are summarised in Table 2. The most frequently reported symptoms that were experienced ‘all’ or ‘most of the time’ included hair loss (*n* = 11/15; 73.3%), sleep disturbance (*n* = 11/16; 68.8%), and joint pain (*n* = 10/18; 55.6%). Muscle weakness (*n* = 8/11; 72.7%), swollen joints (*n* = 10/17; 58.8%), skin rash (*n* = 10/17; 58.8%) and difficulty concentrating (*n* = 7/14; 50.0%) were predominantly attributed to SLE. The symptoms most frequently attributed to LN were urinary frequency (*n* = 6/14; 42.9%), swelling in non-joints (*n* = 7/17; 41.2%) and frequent infections (*n* = 2/5; 40.0%).

**Table 2:** Patient-reported symptoms frequency and attribution to SLE or LN

Symptom category	Symptom description, *n* (%)^1^	Amount of time symptom is experienced, *n* (%)	The type of lupus the symptoms are attributed to, *n* (%)
Pain and discomfort	Joint pain, 18 (90.0)	4 (22.2) All of the time 6 (33.3) Most of the time 8 (44.4) A good bit of the time 0 (0.0) Occasionally 0 (0.0) Never	8 (44.4) SLE 5 (27.8) LN 5 (27.8) Both 0 (0.0) Do not know
	Headaches, 14 (70.0)	0 (0.0) All of the time 2 (14.3) Most of the time 4 (28.6) A good bit of the time 8 (57.1) Occasionally 0 (0.0) Never	3 (21.4) SLE 1 (7.1) LN 4 (28.6) Both 6 (42.9) Do not know
	Back pain, 10 (50.0)	1 (10.0) All of the time 1 (10.0) Most of the time 3 (30.0) A good bit of the time 5 (50.0) Occasionally 0 (0.0) Never	3 (30.0) SLE 2 (20.0) LN 5 (50.0) Both 0 (0.0) Do not know
	Muscle pain, 7 (35.0)	0 (0.0) All of the time 2 (28.6) Most of the time 1 (14.3) A good bit of the time 4 (57.1) Occasionally 0 (0.0) Never	2 (28.6) SLE 2 (28.6) LN 3 (42.9) Both 0 (0.0) Do not know
	Body aches, 2 (10.0)	0 (0.0) All of the time 0 (0.0) Most of the time 2 (100.0) A good bit of the time 0 (0.0) Occasionally 0 (0.0) Never	1 (50.0) SLE 0 (0.0) LN 1 (50.0) Both 0 (0.0) Do not know
Energy-related symptoms	Fatigue or tiredness, 20 (100.0)	3 (15.0) All of the time 4 (20.0) Most of the time 6 (30.0) A good bit of the time 7 (35.0) Occasionally 0 (0.0) Never	6 (30.0) SLE 1 (5.0) LN 9 (45.0) Both 4 (20.0) Do not know
	Muscle weakness, 11 (55.0)	1 (9.1) All of the time 4 (34.4) Most of the time 0 (0.0) A good bit of the time 6 (54.5) Occasionally	8 (72.7) SLE 0 (0.0) LN 1 (9.1) Both 1 (9.1) Do not know
Swelling (in joints and non-joints)	Swollen joints, 17 (85.0)	2 (11.8) All of the time 2 (11.8) Most of the time 6 (35.3) A good bit of the time 7 (41.2) Occasionally 0 (0.0) Never	10 (58.8) SLE 1 (5.9) LN 3 (17.6) Both 3 (17.6) Do not know
	Swelling in hands, legs, or feet (non-joint), 17 (85.0)	5 (29.4) All of the time 2 (11.8) Most of the time 3 (17.6) A good bit of the time 7 (41.2) Occasionally 0 (0.0) Never	4 (23.5) SLE 7 (41.2) LN 4 (23.5) Both 2 (11.8) Do not know
Skin symptoms	Skin rash, 17 (85.0)	2 (11.8) All of the time 1 (5.9) Most of the time 2 (11.8) A good bit of the time 11 (64.7) Occasionally 1 (5.9) Never^2^	10 (58.8) SLE 1 (5.9) LN 2 (11.8) Both 4 (23.5) Do not know
	Skin ulcers (mouth), 6 (30.0)	0 (0.0) All of the time 0 (0.0) Most of the time 1 (16.7) A good bit of the time 4 (66.7) Occasionally 1 (16.7) Never^2^	4 (66.7) SLE 0 (0.0) LN 1 (16.7) Both 1 (16.7) Do not know
	Skin ulcers (extremities), 4 (20.0)	0 (0.0) All of the time 0 (0.0) Most of the time 1 (25.0) A good bit of the time 3 (75.0) Occasionally 0 (0.0) Never	2 (50.0) SLE 1 (25.0) LN 1 (25.0) Both 0 (0.0) Do not know
Cognitive symptoms	Difficulty concentrating, 14 (70.0)	1 (7.1) All of the time 3 (21.4) Most of the time 7 (50.0) A good bit of the time 3 (21.4) Occasionally 0 (0.0) Never	7 (50.0) SLE 0 (0.0) LN 5 (35.7) Both 2 (14.2) Do not know
	Forgetfulness, 13 (65.0)	3 (23.1) All of the time 1 (7.6) Most of the time 3 (23.1) A good bit of the time 6 (46.1) Occasionally 0 (0.0) Never	6 (46.1) SLE 0 (0.0) LN 4 (30.7) Both 3 (23.0) Do not know
Gastrointestinal and digestive symptoms	Nausea, 7 (35.0)	1 (14.2) All of the time 1 (14.2) Most of the time 1 (14.2) A good bit of the time 4 (57.1) Occasionally 0 (0.0) Never	0 (0.0) SLE 1 (14.2) LN 2 (28.5) Both 4 (57.1) Do not know
	Abdominal pain, 5 (25.0)	1 (20.0) All of the time 0 (0.0) Most of the time 1 (20.0) A good bit of the time 3 (60.0) Occasionally 0 (0.0) Never	0 (0.0) SLE 1 (20.0) LN 2 (40.0) Both 2 (40.0) Do not know
	Upset stomach, 4 (20.0)	0 (0.0) All of the time 1 (25.0) Most of the time 1 (25.0) A good bit of the time 2 (50.0) Occasionally 0 (0.0) Never	0 (0.0) SLE 0 (0.0) LN 3 (75.0) Both 1 (25.0) Do not know
	Diarrhoea, 3 (15.0)	1 (33.3) All of the time 0 (0.0) Most of the time 1 (33.3) A good bit of the time 1 (33.3) Occasionally 0 (0.0) Never	1 (33.3) SLE 0 (0.0) LN 1 (33.3) Both 1 (33.3) Do not know
	Vomiting, 3 (15.0)	1 (33.3) All of the time 0 (0.0) Most of the time 0 (0.0) A good bit of the time 0 (0.0) Occasionally 2 (66.6) Never^2^	0 (0.0) SLE 1 (33.3) LN 2 (66.6) Both 0 (0.0) Do not know
Respiratory symptoms	Shortness of breath, 5 (25.0)	1 (20.0) All of the time 1 (20.0) Most of the time 1 (20.0) A good bit of the time 2 (40.0) Occasionally 0 (0.0) Never	2 (40.0) SLE 0 (0.0) LN 1 (20.0) Both 2 (40.0) Do not know
Additional symptoms	Sleep disturbance, 16 (80.0)	5 (31.2) All of the time 6 (37.5) Most of the time 3 (18.7) A good bit of the time 2 (12.5) Occasionally 0 (0.0) Never	4 (25.0) SLE 0 (0.0) LN 8 (50.0) Both 4 (25.0) Do not know
	Hair loss, 15 (75.0)	7 (46.6) All of the time 4 (26.6) Most of the time 3 (20.0) A good bit of the time 0 (0.0) Occasionally 1 (6.7) Never^2^	6 (40.0) SLE 0 (0.0) LN 6 (40.0) Both 3 (20.0) Do not know
	Urinary frequency, 14 (70.0)	4 (28.5) All of the time 3 (23.1) Most of the time 4 (28.5) A good bit of the time 2 (14.2) Occasionally 2 (14.2) Never^2^	0 (0.0) SLE 6 (42.9) LN 5 (35.7) Both 2 (14.3) Do not know
	Weight gain, 14 (70.0)	5 (35.7) All of the time 0 (0.0) Most of the time 0 (0.0) A good bit of the time 5 (35.7) Occasionally 4 (28.5) Never^2^	0 (0.0) SLE 2 (14.2) LN 6 (42.9) Both 6 (42.9) Do not know
	Fever, 9 (45.0)	2 (22.2) All of the time 1 (12.5) Most of the time 2 (22.2) A good bit of the time 4 (37.5) Occasionally 0 (0.0) Never	2 (22.2) SLE 1 (12.5) LN 3 (37.5) Both 2 (22.2) Do not know
	Moon face, 9 (45.0)	3 (33.3) All of the time 0 (0.0) Most of the time 0 (0.0) A good bit of the time 6 (66.6) Occasionally 0 (0.0) Never	3 (33.3) SLE 0 (0.0) LN 2 (22.2) Both 3 (33.3) Do not know
	Frequent infections, 5 (25.0)	2 (40.0) All of the time 0 (0.0) Most of the time 0 (0.0) A good bit of the time 3 (60.0) Occasionally 0 (0.0) Never	0 (0.0) SLE 2 (40.0) LN 1 (20.0) Both 2 (40.0) Do not know
	Purple fingertips, 4 (20.0)	0 (0.0) All of the time 1 (25.0) Most of the time 1 (25.0) A good bit of the time 2 (50.0) Occasionally 0 (0.0) Never	1 (25.0) SLE 0 (0.0) LN 1 (25.0) Both 2 (50.0) Do not know

^1^n represents number of patients reporting symptom; ^2^patient had experienced the symptom before but not currently

*LN* lupus nephritis; *SLE* systemic lupus erythematosus

All patients described symptoms within the pain and discomfort subdomain, the largest of the identified domains for reported symptom expressions (Supplementary Table 1). Within this subdomain, the most commonly expressed symptom concept was joint pain.

*“*… *It hurts. Sometimes it hurts so bad like I’m crying. It gets really*,* really bad.”*

*“Mostly it’s just the pain is so excruciating, I can’t – I’m in tears.”*

*“I had a lot of joint pain. I wasn’t able to walk.”*

Energy-related symptoms were also experienced by all patients (Supplementary Table 1). Within this symptom subdomain, fatigue was the most commonly reported symptom. Patients described having no energy for anything, especially during flares; some patients stated that their tiredness was not relieved after sleep or rest. Muscle weakness and tiredness were reported even after light chores.

“I am tired most of the time…I pretty much wake up tired, I’m tired throughout the day, and I go to bed tired.”

Other common symptom subdomains included skin symptoms and gastrointestinal and digestive symptoms, which were experienced by almost all patients (Supplementary Table 1). Skin rash was the most commonly expressed symptom within the skin symptom subdomain.

*“My entire body was covered in…a rash*,* it was red splotches all over my body*,* mostly my arms*,* my legs*,* and my face area*,* and chest.”*

Almost all patients also experienced symptoms within the swelling and cognitive subdomains. Joint and non-joint swellings were the most commonly expressed symptoms within the swelling subdomain, and forgetfulness and difficulty concentrating within the cognitive subdomain. Almost half of patients experienced respiratory symptoms, with difficulty breathing being the most common. There were many symptoms in the ‘additional symptoms’ subdomain; the most commonly reported symptoms included urinary, hair loss, and weight gain (Supplementary Table 1), indicating the broad range of symptoms experienced by patients.

Symptom severity and bothersomeness are summarised in Table 3. Patients reported shortness of breath as the most severe symptom at its worst (*n* = 5, NRS rating = 10.00), followed by swollen joints (*n* = 17, NRS rating = 8.53) and sleep disturbance (*n* = 16, NRS rating = 8.33). The most bothersome symptoms expressed by patients included shortness of breath (*n* = 5, NRS rating = 10.00), back pain (*n* = 10, NRS rating = 8.00), body aches (*n* = 2, NRS rating = 8.00), sleep disturbance (*n* = 16, NRS rating = 7.93), physical fatigue (*n* = 20, NRS rating = 7.90), and joint pain (*n* = 18, NRS rating = 7.89).

**Table 3:** Symptom severity and bothersomeness, ranked within categories by bothersomeness

Symptom category	Symptom description	0 to 10 point scale; 0 = not severe/bothersome, 10 = extremely severe/bothersome		
		Number of patients who rated symptom, *n* (%)	Severity of symptom at its worst, mean (SD)	Bothersomeness of symptom, mean (SD)
Pain and discomfort	Joint pain	18 (90.0)	8.28 (1.53)	7.89 (2.19)
	Headaches	14 (70.0)	7.14 (2.14)	7.29 (1.89)
	Back pain	10 (50.0)	7.89 (1.73)	8.00 (3.74)
	Muscle pain	7 (35.0)	7.29 (2.29)	7.43 (2.94)
	Body aches	2 (10.0)	7.50 (0.71)	8.00 (0.00)
Energy-related symptoms	Physical fatigue	20 (100.0)	7.85 (1.50)	7.90 (1.74)
	Mental fatigue	13 (65.0)	7.23 (2.35)	6.69 (2.66)
	Muscle weakness	11 (55.5)	6.45 (2.66)	6.18 (2.89)
Swelling (joints and non-joints)	Swollen joints	17 (85.0)	8.53 (1.49)	7.76 (2.17)
	Swollen hands, legs, or feet (non-joint)	17 (85.0)	7.71 (2.14)	6.53 (2.96)
Skin symptoms	Skin rash	17 (85.0)	6.13 (2.45)	5.50 (3.01)
	Skin ulcers (mouth)	6 (30.0)	6.00 (2.12)	6.20 (2.28)
	Skin ulcers (extremities)	4 (20.0)	4.25 (1.26)	2.00 (1.51)
Cognitive symptoms	Difficulty concentrating	14 (70.0)	6.21 (2.04)	6.79 (2.15)
	Forgetfulness	13 (65.0)	6.38 (2.15)	7.15 (2.91)
Gastrointestinal and digestive symptoms	Nausea	7 (35.0)	4.83 (2.48)	4.50 (2.95)
	Abdominal pain	5 (25.0)	8.40 (1.52)	7.20 (2.28)
	Upset stomach	4 (20.0)	7.67 (2.08)	7.67 (2.52)
	Diarrhoea	3 (15.0)	7.00 (1.00)	5.67 (1.53)
	Vomiting	3 (15.0)	6.50 (4.73)	5.50 (4.12)
Respiratory symptoms	Shortness of breath	5 (25.0)	10.00 (0.00)	10.00 (0.00)
Additional symptoms	Sleep disturbance	16 (80.0)	8.33 (1.72)	7.93 (1.94)
	Hair loss	15 (75.0)	7.79 (3.04)	7.14 (3.59)
	Urinary frequency	14 (70.0)	7.33 (2.64)	7.75 (2.96)
	Weight gain	14 (70.0)	7.42 (2.71)	7.50 (3.15)
	Fever	9 (45.0)	7.20 (2.94)	6.00 (3.16)
	Moon face	9 (45.0)	6.33 (2.45)	4.67 (3.81)
	Frequent infections	5 (25.0)	7.20 (1.30)	7.40 (2.97)
	Purple fingertips	4 (25.0)	8.00 (2.83)	7.50 (3.54)

*SD* standard deviation

### Impact of LN

Patients expressed that LN significantly impacted their lives. All patients reported changes in daily performance and lifestyle (Table 4). The most frequently impacted areas of daily life included work, physical activity, and everyday activities. Additionally, patients expressed trouble focusing on school or having to take a lot of time off work. Some patients reported that their disease affected their career choices. Patients also reported that they could no longer participate in physical activities, impacting their ability to maintain a healthy weight.

**Table 4:** Patient-reported impact subdomains

Patient-reported impact subdomains	Number of patient language expressions within concept	Percentage of total symptom expressions (*n* = 318), %	Number of transcripts contributing to concept expression (*n* = 20)	Percentage of transcripts contributing to concept expression, %
**Changes in daily performance and lifestyle**	122	38.4	N/A	N/A
Work	39	12.3	16	80.0
Physical activity	25	7.9	12	60.0
Everyday activities	24	7.5	16	80.0
Mobility	16	5.0	6	30.0
Leisure	9	2.8	5	25.0
School	6	1.9	3	15.0
Diet	2	0.6	1	5.0
Need to rest more	1	0.3	1	5.0
**Impacts on relationships and social functioning**	60	18.9	N/A	N/A
Social activities	23	7.2	12	60.0
Family	21	6.6	7	35.0
Friends	11	3.5	8	40.0
Partner/spouse	5	1.6	2	10.0
**Emotional health**	73	23.0	N/A	N/A
Anxiety	19	6.0	14	70.0
Worry	19	6.0	14	70.0
Sadness/depression	16	5.0	11	55.0
Low motivation	6	1.9	1	5.0
Low self-confidence	8	2.5	7	35.0
Frustration	2	0.6	1	5.0
Embarrassed	1	0.3	1	5.0
Stress	1	0.3	1	5.0
Cranky	1	0.3	1	5.0
**Sleep disturbances**	35	11.0	N/A	N/A
Reduced sleep quality	15	4.7	8	40.0
Difficulty falling asleep	12	3.8	8	40.0
Difficulty staying asleep	7	2.2	7	35.0
Sleep disturbance	1	0.3	1	5.0
**Additional impacts**	28	8.8	N/A	N/A
Treatment burden	24	7.5	6	30.0
Examine own body	1	0.3	1	5.0
Pregnancy	1	0.3	1	5.0
Quality of life	1	0.3	1	5.0
Waking up	1	0.3	1	5.0

*N/A* not applicable

“I can’t really go to school full time or work full time because of the fatigue.”

“I don’t do as many of the things…I could do, I run out of steam…I can’t do two days of weekly activities back-to-back [due to fatigue].”

LN also negatively affected patients’ emotional health, with anxiety and worry being the most commonly reported symptoms of this subdomain. One patient described her anxiety as *“Just always just feeling completely drained. It just takes a toll on you mentally and it brings you down and gives you some anxiety.”*

Many patients reported their SLE and LN had impacts on their relationships and social functioning, with ‘social activities’ having the greatest number of expressions. Patients described that they avoid social activities due to lack of energy or anxiety and that they are unable to spend quality time with their families and friends. Patients emphasised that their disease puts strain on their relationships as they often require help with day-to-day tasks.

“I don’t hang out with any people…I don’t…surround myself with anyone.”

“If I’m not able to spend time with loved ones because I’m not feeling well, that bothers me.”

### Treatment experience and treatment satisfaction

Nearly all patients (*n* = 19) were satisfied with their current treatment, with a mean (SD) NRS score of 8.11 (1.91). With current medications, patients reported reductions in inflammation and flares, improvements in mobility, reductions in joint pain and swelling, as well as an overall improvement in kidney function.

“Well, I have experienced an improvement from the last major problem I had, which was the nephritis. My kidneys are in remission, nephritis is under control, lupus is under control at the moment…and I’m not having flares, that’s a big goal.”

However, patients also said they were hoping to see more improvements in their kidney and lung symptoms, as well as improvements in fatigue and skin rash.

“Skin rash to improve or go away…the fatigue not to be as severe or frequent.”

Patient expectations for long-term treatment goals included a reduction in medication burden and a reduction in pain.

*“That I can feel better without feeling the same all day…I want to be in less pain.”*

In addition, patients reported concerns over their fertility.

“I am hoping that it doesn’t turn into really bad arthritis in the future, so immobility, infertility. Those are my two main concerns.”

### Cognitive interviews

A total of 10 patients responded to cognitive interview questions for the FACIT-Fatigue and another 10 responded to cognitive interview questions for the LupusQoL. The questions asked in the FACIT-Fatigue interview were deemed by the patients to be relevant to their experience with SLE and/or LN, indicating content validity. Overall, patients stated that the instructions were understandable, all items were easily understood, and they were able to answer easily using the provided response options.

Feedback and suggestions pertained to some general clarifications, including one patient who did not understand that their response should have been for the last 7 days but instead recalled their experience since their LN diagnosis. Recommendations for future use of the measures included appropriate patient orientation to ensure patients understand to answer the items within the 7-day time frame, even when items are relevant to their experience over a longer period of time. Patients also recommended providing clarity regarding the definitions of items 1–4 in the FACIT-Fatigue (“I feel fatigued”, “I feel weak all over”, “I feel listless [“washed out”]”, “I feel tired”) as they were perceived to address similar concepts. Additional suggestions for future use of these measures might include clarity about these terms as part of the orientation of patients to the PRO before it is carried out.

The questions asked in the LupusQoL interview were also deemed by the patients to be relevant to their experience with SLE and/or LN, indicating content validity. Overall, patients stated that the instructions were understandable, all items were easily understood, and they were able to answer easily using the provided response options. Not all symptoms were currently experienced (e.g., hair loss, weight gain or issues with concentrating), but they had been experienced in the past and were still felt to be relevant.

As with FACIT-Fatigue, feedback on LupusQoL related to general clarifications rather than aspects of the disease. For instance, clarity about the recall period of the last 4 weeks may need to be provided when first applying the measure. Several patients struggled to understand whether ‘never’ meant never during the past 4 weeks or never at all (not ever).

## Discussion

This study evaluated the content validity of FACIT-Fatigue and Lupus QoL in patients with LN. This study demonstrates that pain, discomfort and fatigue (e.g., joint pain, gastrointestinal issues, tiredness and fatigue), cognitive symptoms (e.g., difficulty in concentrating and forgetfulness), sleep-related issues and skin rashes are prevalent in patients with LN, consistent the overall SLE population [16, 33, 34]. This study also explored characteristics of the symptoms (i.e., severity, frequency, bothersomeness) and impacts that provide additional information around the burden experienced by patients with LN, expanding on evidence gathered in a previous qualitative interview study [35].

Although patients were often unable to attribute symptoms specifically to SLE or LN, some symptoms (e.g., muscle weakness and mouth ulcers) were most frequently attributed to SLE, while non-joint swelling and frequent infections were most frequently attributed to LN. The symptoms rated with the highest level of severity were shortness of breath, followed by swollen joints and sleep disturbances. The most bothersome symptoms reported by patients included shortness of breath, back pain, and body aches.

The interviews also revealed that symptoms and impacts of LN result in a high burden on patients’ daily lifestyle, emotional health and HRQoL. This study identified symptoms and impacts that, if eased, might best address patients’ long-term unmet needs, such as reducing medication burden and pain, though concerns about fertility remain. Overall, patients were generally satisfied with their current treatment, though they felt improvements were needed to provide satisfactory relief when symptoms were at their worst.

The inter-rater agreement was high between coders for both the identification of concepts and the allocation of codes, suggesting good consistency between coders [31]. While saturation of concept was technically not achieved, the additional symptoms identified were closely related to already mentioned concepts but were expressed using more specific language (e.g., skin infections and scars are both skin symptoms). Therefore, the additional appearance of more specific symptom descriptions was not particularly worrisome to the overall data quality. Furthermore, the appearance of similar but more detailed symptoms suggested a high level of comprehensiveness. Patients did not report having additional symptoms that were not covered by the content of the measures. These results generate confidence that the measures reflected the most important and relevant symptoms experienced by patients with LN.

Patients in this study reported that the FACIT-Fatigue and LupusQoL measures were relevant to their experience of SLE/LN and the items and response options were well understood. This is consistent with another recent study, which also showed that symptoms and impact concepts assessed in these PRO measures were well understood and relevant in patients with LN [35]. However, there was some inconsistency in the recall period used by patients considering their symptoms. As a result, a recommendation from the study was to ensure that patient training related to completing the PROs included a reminder to consider the appropriate recall periods when patients are first asked to complete the measure. Furthermore, while no actions were indicated in the wording or structure of the response scale for either questionnaire, definitions are needed to provide clarity to patients on how to view the items that are often perceived as similar concepts, such as fatigue and tiredness. Additionally, an important recommendation emerged regarding how patients should respond to items that may be relevant to them overall but were not experienced within the specified recall period used by the measure. Although this recommendation was mentioned by only one patient during the FACIT-Fatigue interview, it suggests a potential issue that may affect a larger study population. Thus, future researchers should consider providing instructions on how to handle such situations to ensure accurate and meaningful responses are provided by patients.

While LupusQoL and FACIT-Fatigue have been validated in the overall SLE population [15–18], they have not yet been validated in an SLE population that also has LN. Therefore, the findings of this study provide insights for both researchers and clinicians seeking to use LupusQoL and FACIT-Fatigue in patients with LN. However, further psychometric validation is needed to establish construct validity of both measures for use in the LN patient population.

Further analyses are warranted to examine the relationship between the severity and bothersomeness of these symptoms in patients with LN versus those with non-nephritis SLE, and to examine the symptom burden and lived experience of patients with lupus nephritis.

As a limitation, the main study did not reach the target sample size due to the coronavirus pandemic, which may have contributed to a more limited spectrum of LN amongst enrolled patients in the qualitative sub-study. Only two patients were considered to have active LN, and two patients were receiving induction therapy at the time of study screening. Difficulties with recruiting patients with active LN meant that purposive sampling was not possible, highlighting a challenge in recruiting participants for studies investigating health states with an acute nature. The primary study used purposive sampling to identify participants, and interviews of this sub-study were conducted with those who agreed to participate from this sample; it is commonly understood that research study samples do not always reflect the broader population. Despite these limitations, this study adds to the limited qualitative literature exploring key symptoms and HRQoL impact concepts important to patients with LN, confirming overlap in experiences and appropriateness of assessing the same concepts already identified by qualitative research in SLE [13, 16, 33]. These patient perspectives provide important information regarding patient perceptions of symptom burdens as part of their broader experience.

## Conclusions

This study provides the necessary evidence supporting the content validity of the FACIT-Fatigue and LupusQoL measures for assessing fatigue- and quality of life–related concepts in patients with LN. Concept elicitation results show that the content of the items is relevant to the patient descriptions of their symptoms and impact experience with LN. The results of the cognitive interviews show that patients appropriately understand the content of these measures. No additional symptoms were suggested by patients as important concepts to include, suggesting comprehensiveness of the content of the measures. While some minor issues with recall periods and unique definitions of similar concepts arose during the interviews, these are standard issues found in the administration process of most outcome measures and not specific to the condition of LN. These are easily managed for SLE and LN patients alike, with more attention to the patient’s orientation to the measure when it is first completed.

## Electronic supplementary material

Below is the link to the electronic supplementary material.

Supplementary Material 1

## Data Availability

Anonymised individual participant data and study documents can be requested for further research from https://www.gsk-studyregister.com/en/.

## Abbreviations

FACIT-Fatigue: Functional Assessment of Chronic Illness Therapy-Fatigue
HRQoL: Health-related quality of life
IQR: Interquartile range
LN: Lupus nephritis
LupusQoL: Lupus Quality of Life
NRS: Numerical rating scale
PRO: Patient-reported outcome
SD: Standard deviation
SF-36: 36-item Short Form Survey
SLE: Systemic lupus erythematosus
