# Supplementary material for: Patient-reported outcome measures for lupus nephritis: content validity of LupusQoL and FACIT-Fatigue
Source: J Patient Rep Outcomes. 2024 Sep 30;8:115. doi: 10.1186/s41687-024-00783-z (PMC11442872; doi:10.1186/s41687-024-00783-z)
Supplement: Supplementary file 1 — Supplementary Material 1 [file 41687_2024_783_MOESM1_ESM.pdf]

SUPPLEMENTARY MATERIALS

MAIN STUDY: METHODS

This was a non-interventional, multicentre, patient-completed, cross-sectional survey study with a retrospective chart review component, carried out between December 2020 and November 2021 across 15 sites in the USA, Germany, Canada, and Austria. The primary objective was to describe the health-related quality of life (HRQoL) of patients with lupus nephritis (LN), as assessed by the 36-item Short Form Survey version 2 (SF-36v2). The secondary objectives included the description of the disease-specific HRQoL of patients with LN, assessed by the Lupus Quality of Life Questionnaire (LupusQoL), and fatigue, assessed by Functional Assessment of Chronic Illness Therapy-Fatigue (FACIT-Fatigue).

Patient identification and eligibility criteria

Site staff screened patients' medical charts to identify a preliminary set of eligible patients. Patients eligible for the study were adults (≥18 years of age) with systemic lupus erythematosus (SLE) and biopsy-proven LN (Class III–VI [with or without Class V] or pure Class V). Patients presenting with suspected LN after 1 March 2020, in whom a biopsy was not possible owing to the COVID-19 pandemic, were also eligible if the investigator found that they had proliferative/membranous LN (from clinical/laboratory findings) requiring immunosuppressive therapy. Eligible patients had to have ≥2 assessments of the following renal laboratory test results from the medical record within the last 6 months, with the most recent assessment within 3 months from screening: urinary protein:creatinine ratio (uPCR; or 24-hour proteinuria or albumin:creatinine ratio), urine sediment (activity), or serum creatinine or estimated glomerular filtration rate (eGFR); or measured GFR if eGFR was not available). Patients were excluded if they were enrolled in any interventional clinical trial.

The identified eligible patients were contacted by trained staff, who provided an overview of the study. To be enrolled, patients were required to confirm study interest, understand the requirements of the study and provide informed consent (including consent for the use and disclosure of research-related health information as well as to medical record access), have a functional email address and be able to use a computer or handheld device with internet access, speak and read English, Spanish or German, and complete a one-time 30- to 40-minute electronic survey within 7 calendar days of enrolment.

Data source and collection

Data were collected cross-sectionally from the one-time patient electronic survey and retrospectively from patients' medical records.

The patient surveys were completed in the following order and skipping ahead was not possible: demographic characteristics, SF-36v2, LupusQoL, FACIT-Fatigue, patient global assessment, current treatment satisfaction 7-point Likert scale, Work Productivity and Activity Impairment (WPAI-Lupus), and European Quality of Life 5 Dimensions 3 Level Version.

MAIN STUDY: RESULTS

Patient population

Data collection was stopped early owing to poor recruitment and challenges completing the study objectives in a reasonable time frame during the COVID-19 pandemic. In total, 83 eligible patients were enrolled (65 [78.3%] from the USA, 6 (7.2%) from Canada, 8 (9.6%) from Germany, 4 (4.8%) from Austria); 44 completed all questionnaires (USA, 33 [75.0%]; Canada, 4 [9.1%]; Germany, 5 [11.4%]; Austria, 2 [4.5%]) and had their charts reviewed. Most patients were female (42 [95.5%]), and the mean (standard deviation, SD) age of patients at enrolment was 37 (11.5) years

(**Supplementary Table 2**). The median (interquartile range, IQR) duration since SLE and LN diagnosis to the consent date were 11.8 (6.3, 19.7) and 5.2 (3.2, 11.1) years, respectively.

HRQoL of patients with LN

Mean (SD) physical component summary (PCS) and mental component summary (MCS) scores of the SF-36v2 were 45.8 (9.7) and 45.6 (11.2), respectively. Mean domain scores were below 50. The domain with the lowest mean (SD) score was general health perceptions (40.5 [10.1]), followed by social functioning (45.0 [11.0]) and role limitations due to emotional problems (45.2 [10.4]) (**Supplementary Figure 1**). The bodily pain domain showed the highest mean score (48.1 [10.4]), followed by mental health (47.0 [11.5]) and physical functioning (46.9 [10.6]).

The mean (SD) FACIT-Fatigue score was 32.9 (13.3). The mean (SD) total scores by LupusQoL domain were 73.3 (24.4) in physical health, 71.4 (27.9) in pain, 75.2 (32.4) in planning, 76.4 (29.6) in intimate relationships, 58.0 (32.4) in burden to others, 73.5 (26.0) in emotional health, 72.5 (28.8) in body image, and 59.8 (28.4) in fatigue.

SUPPLEMENTARY TABLES AND FIGURES

Supplementary Table 1. Patient-reported symptom subdomains and concepts

Symptom subdomains	Number of patient language expressions within concept, n	Percentage of total symptom expressions, % N=670	Patients expressing concept, n (%) N=20
**Pain and discomfort**	**151**	**22.5**	**20 (100)**
Joint pain	68	10.1	17 (85.0)
Headache	21	3.1	13 (65.0)
Pain (otherwise unspecified)	30	4.5	11 (55.0)
Back pain	11	1.6	9 (45.0)
Muscle pain	17	2.5	7 (35.0)
Body aches	2	0.3	2 (10.0)
Neuropathy	2	0.3	1 (5.0)
**Energy-related symptoms**	**125**	**18.7**	**20 (100)**
Tiredness	48	7.2	17 (85.0)
Fatigue	56	8.4	16 (80.0)
Muscle weakness	12	1.8	9 (45.0)
Decreased energy	7	1.0	5 (25.0)
Weakness (general)	2	0.3	2 (10.0)
**Skin symptoms**	**72**	**10.7**	**19 (95.0)**
Skin rash	44	6.6	16 (80.0)
Skin ulcers	15	2.2	11 (55.0)
Itchy skin	6	0.9	2 (10.0)
Irritated skin	2	0.3	2 (10.0)
Cuts	1	0.1	1 (5.0)
Discoid scars	1	0.1	1 (5.0)
Skin infection	1	0.1	1 (5.0)

Skin scar	1	0.1	1 (5.0)
Stretched skin	1	0.1	1 (5.0)
**Gastrointestinal and digestive symptoms**	**64**	**9.6**	**19 (95.0)**
Nausea	11	1.6	6 (30.0)
Abdominal pain	7	1.0	4 (20.0)
Vomiting	6	0.9	4 (20.0)
Decreased appetite	4	0.6	4 (20.0)
Diarrhoea	4	0.6	3 (15.0)
Upset stomach	3	0.4	3 (15.0)
Acid reflux	2	0.3	2 (10.0)
Bloated	2	0.3	2 (10.0)
Choking	1	0.1	1 (5.0)
Constipation	1	0.1	1 (5.0)
**Swelling (joint and non-joint)**	**59**	**8.8**	**18 (90.0)**
Joint swelling	33	4.9	14 (70.0)
Swelling (not joints)	25	3.7	11 (55.0)
Water retention	1	0.1	1 (5.0)
**Cognitive symptoms**	**37**	**5.5**	**18 (90.0)**
Forgetfulness	19	2.8	13 (65.0)
Difficulty concentrating	15	2.2	10 (50.0)
Brain fog	3	0.4	3 (15.0)
**Respiratory symptoms**	**29**	**4.3**	**9 (45.0)**
Difficulty breathing	23	3.4	8 (40.0)
Respiratory infections	2	0.3	2 (10.0)
General respiratory issues	2	0.3	1 (5.0)
Cough	1	0.1	1 (5.0)
Fluid in lungs	1	0.1	1 (5.0)
**Additional symptoms**	**133**	**19.9**	**20 (100)**

Urinary symptoms	32	4.8	15 (75.0)
Weight gain	23	3.4	15 (75.0)
Hair loss	29	4.3	14 (70.0)
Fever	15	2.2	8 (40.0)
Moon face	12	1.8	10 (50.0)
Eyesight changes	10	1.5	1 (5.0)
Raynaud's Phenomenon	7	1.0	4 (20.0)
Infections	5	0.7	5 (25.0)
Stiffness	5	0.7	4 (20.0)
High blood pressure	4	0.6	2 (10.0)
Bruises	3	0.4	2 (10.0)
Photosensitivity	2	0.3	2 (10.0)
White film in mouth	1	0.1	2 (10.0)
Blood clot	1	0.1	1 (5.0)
Changing joints	1	0.1	1 (5.0)
Chills	1	0.1	1 (5.0)
Episcleritis	1	0.1	1 (5.0)
Increased heart rate	1	0.1	1 (5.0)
Inflammation	1	0.1	1 (5.0)
Seizures	1	0.1	1 (5.0)
Sore throat	1	0.1	1 (5.0)

Supplementary Table 2. Patient demographics and clinical characteristics of the overall population of the main study

	**N=44**
**Age (years), mean (SD)**	37.0 (11.5)
**Female, n (%)**	42 (95.5)
**Race, n (%)**	
White	10 (22.7)
Black	16 (36.4)
Asian	3 (6.8)
Unknown	1 (2.3)
Other	5 (11.4)
Prefer not to answer	3 (6.8)
Missing[1]	6 (13.6)
**Ethnicity, n (%)**	
Hispanic or Latino	8 (18.2)
Not Hispanic or Latino	24 (54.5)
Prefer not to answer	1 (2.3)
Missing[1]	11 (25.0)
**Education level, n (%)**	
Primary	1 (2.3)
High school	10 (22.7)
College or university	20 (45.5)
Graduate school	12 (27.3)
None	0
Prefer not to say	1 (2.3)
Missing[1]	0
**Age at LN diagnosis (years), mean (SD)**	29.7 (10.9)
**Time since SLE diagnosis (years), median (IQR)[2]**	11.8 (6.3, 19.7)
**Time since LN diagnosis (years), median (IQR)[2]**	5.2 (3.2, 11.1)
**Proteinuria level ≥1 g/day or equivalent by uPCR, n (%)[3]**	8 (18.2)
**Experiencing a renal flare at enrolment, n (%)**	3 (6.8)
**Renal function stable at enrolment, n (%)**	40 (90.9)
**Treatment phase, n (%)[3]**	
Induction of renal response with IV CYC or MMF/MPA	8 (18.2)
Maintenance of renal response	30 (68.2)
Other/none of the above	6 (13.6)
**LN classification, n (%)**	
Class III	11 (25.0)
Class III+V	4 (9.1)
Class IV	16 (36.4)

Class IV+V	6 (13.6)
Class V	7 (15.9)
Class VI (+/- V)	0

[1]Missing values were included in denominator for summarising a variable's distribution; [2]consent date/file date could not be pulled from the EDC system for 7 patients, and the baseline date assigned by data vendor is used to replace data for a given variable; [3]documentation available at patient's most recent visit, as reported by the physician during patient screening.

CYC, cyclophosphamide; EDC, electronic data capture; IQR, interquartile range; IV, intravenous; LN, lupus nephritis; MMF, mycophenolate mofetil; MPA, mycophenolic acid; SD, standard deviation; SLE, systemic lupus erythematosus; uPCR, urinary protein:creatinine ratio.

Supplementary Figure 1. Mean SF-36v2 score by domain for the overall population of the main study

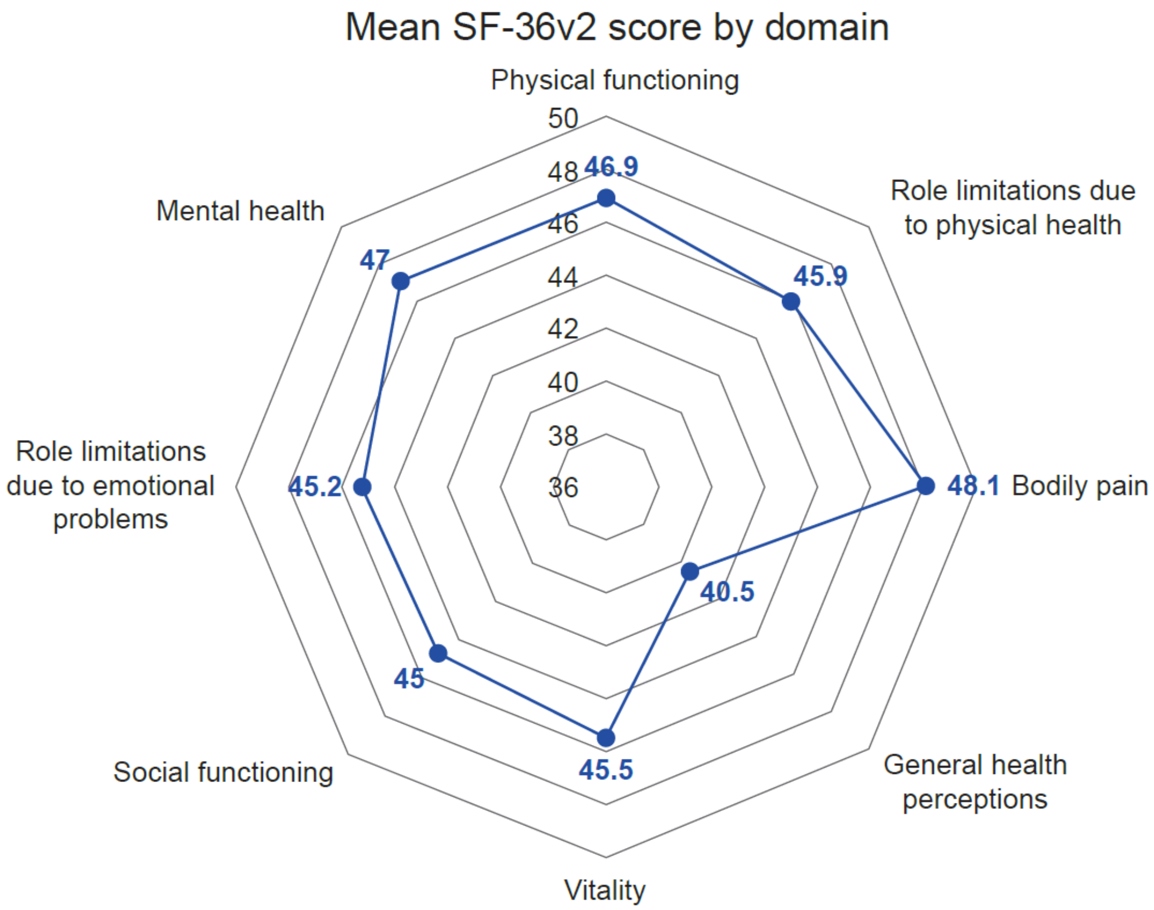

SF-36v2, 36-item Short Form Survey version 2.
